# Addressing significant challenges for animal detection in camera trap images: a novel deep learning-based approach

**DOI:** 10.1038/s41598-025-90249-z

**Published:** 2025-05-09

**Authors:** Margarita Mulero-Pázmány, Sandro Hurtado, Cristóbal Barba-González, María Luisa Antequera-Gómez, Francisco Díaz-Ruiz, Raimundo Real, Ismael Navas-Delgado, José F. Aldana-Montes

**Affiliations:** 1 Department of Animal Biology, University of Málaga, 29071 Málaga, Spain; 2 KHAOS Research Group, ITIS Software, University of Málaga, 29071 Málaga, Spain

**Keywords:** Camera traps, Deep learning, Animal identification, YOLO, Biodiversity, Conservation biology, Computational science

## Abstract

Wildlife biologists increasingly use camera traps for monitoring animal populations. However, manually sifting through the collected images is expensive and time-consuming. Current deep learning studies for camera trap images do not adequately tackle real-world challenges such as imbalances between animal and empty images, distinguishing similar species, and the impact of backgrounds on species identification, limiting the models’ applicability in new locations. Here, we present a novel two-stage deep learning framework. First, we train a global deep-learning model using all animal species in the dataset. Then, an agglomerative clustering algorithm groups animals based on their appearance. Subsequently, we train a specialized deep-learning expert model for each animal group to detect similar features. This approach leverages Transfer Learning from the MegaDetectorV5 (YOLOv5 version) model, already pre-trained on various animal species and ecosystems. Our two-stage deep learning pipeline uses the global model to redirect images to the appropriate expert models for final classification. We validated this strategy using 1.3 million images from 91 camera traps encompassing 24 mammal species and used 120,000 images for testing, achieving an F1-Score of 96.2% using expert models for final classification. This method surpasses existing deep learning models, demonstrating improved precision and effectiveness in automated wildlife detection.

## Introduction

Camera traps are increasingly used as an effective tool to monitor animal populations in ecological research and conservation^1^. These automatically triggered sensors collect images that can be used to conduct fauna inventories, detect elusive species, assess activity patterns, evaluate habitat preferences, estimate occupancy, relative abundance, and density, and serve for management and dissemination purposes^2^. Camera traps have become popular in field biology because they allow studying animals remotely and are minimally invasive compared to traditional capture or marking techniques^3^. Furthermore, camera traps are often described as a low-cost technology because suitable units that gather thousands of images can be purchased for around USD200-800 and only require a few periodic field visits to replace memory cards and batteries, which is generally affordable for wildlife research projects. But the processing of the collected images, which includes visual identification of the targets in the images, labeling, and metadata extraction-and that ultimately leads to data ready for analysis- constitutes a bottleneck that substantially adds up to the cost of the method and results in a considerable delay in data availability^4^. There are programs to facilitate this task, e.g., for labeling the pictures (e.g., DigiKam (https://www.digikam.org/), Agouti (https://agouti.eu/)) or extracting images’ metadata into datasets (e.g., camtrapR^5^) (see^6^ for a review), but despite these advances, visually identifying the species in the images remains a time-consuming, resource-demanding and tedious task.

**Fig. 1:**
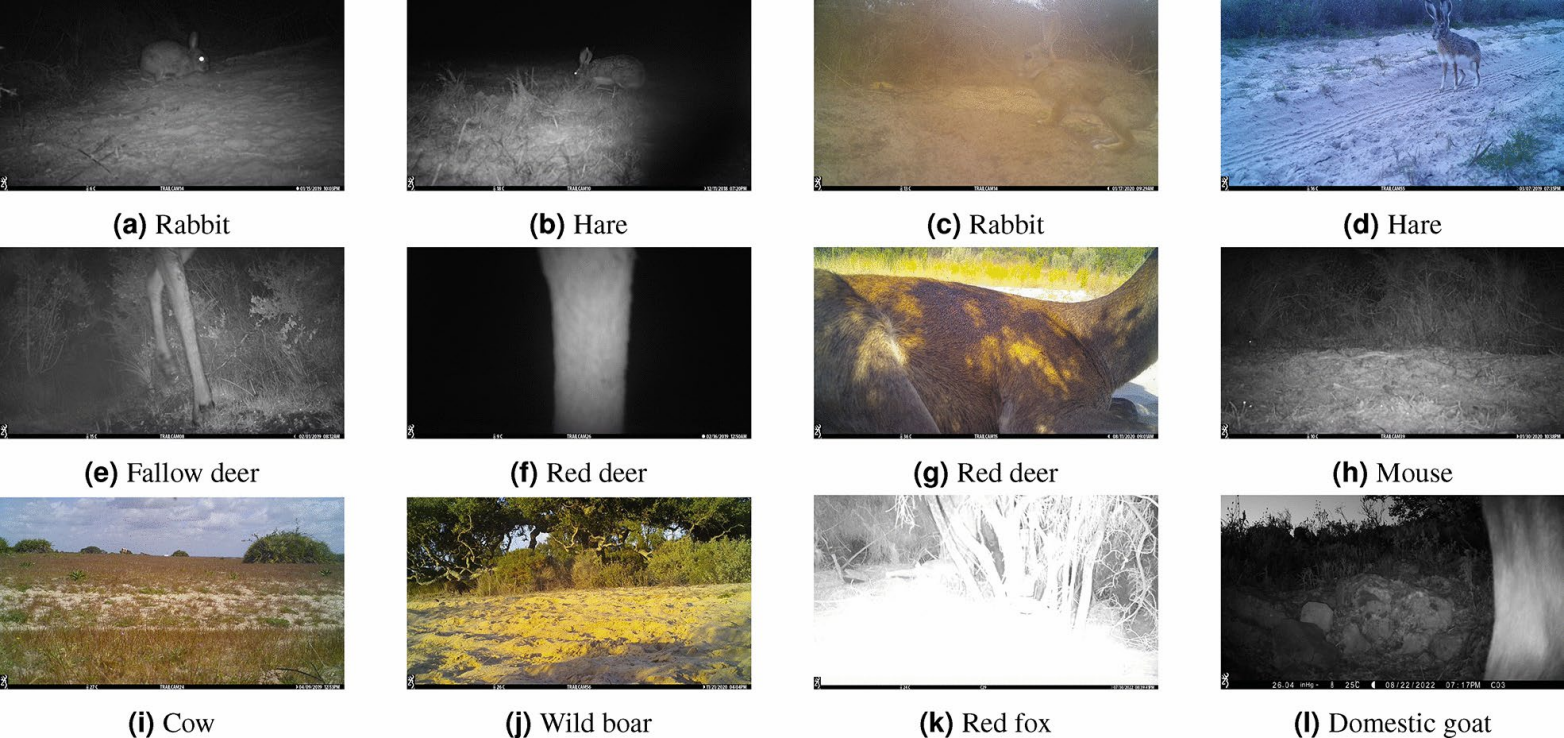
Examples of challenging animal detection in camera trap images. This image illustrates the complexity of differentiating between similar species, such as red deer and fallow deer or rabbits and hares, and detecting animals like small rodents in environments with sparse animal presence. Additionally, it includes scenarios where animals are located very far from or close to the camera and hidden in the background.

In this regard, developing Artificial Intelligence (AI) models for automated species identification represents a dynamic area of ongoing research. Various recent initiatives aim to train AI models using extensive and diverse image datasets^7^. Some of these initiatives also offer easy-to-use software platforms and interfaces to streamline the use of AI^8–11^. However, identifying animals in camera trap images involves difficulties for humans and automated systems that stem from factors such as animals too close to the cameras, partially obscured within the images, or subjected to variations in lighting, shadows, and weather conditions that further complicate the extraction of pertinent information and generate false positives or false negatives in animal detection^12^, as shown in Fig. 1. More specifically the most significant challenges for automating animal detection in camera trap projects include:

- The imbalance between animal and background: The sparse representation of animal samples results in an imbalance between the presence of animals and the background. Empty images often result from cameras being triggered by wind moving surrounding vegetation or due to animals passing by too fast. Consequently, a critical task is to detect whether an image contains an animal (animal vs. empty).
- Minimizing background influence on species identification: The risk of background characteristics affects species identification, for instance, if all wolves consistently appear against a snowy background. In this context, it is essential to reduce the influence of background features on the precise classification of animal species, thereby preventing misclassifications based on shared background characteristics.
- The imbalance between animal species: Camera trap projects often suffer from significant class imbalance^13^, wherein certain species are significantly more prevalent than others. The performance of AI approaches may suffer when models are developed using unbalanced training datasets^14^.
- Differentiating between highly similar animal species: Distinguishing between closely related animal species (E.g. red deer and fallow deer in Fig. 1) can be challenging, mainly when only a tiny portion of the body, such as a leg, is visible. This challenge persists even for expert biologists conducting visual identification.

**Fig. 2:**
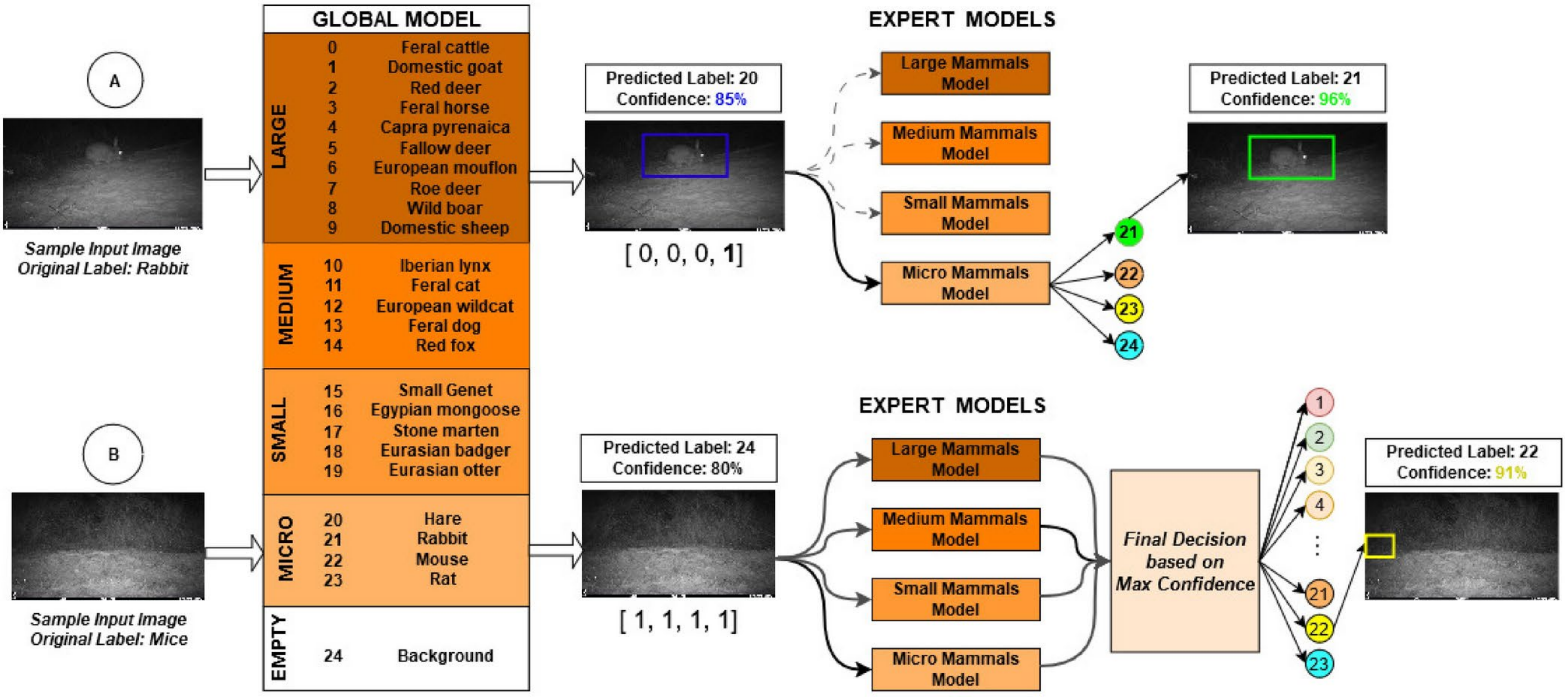
An overview of the proposed methodology, which improves upon the accuracy of a single global detection model trained on all animal classes through a multi-tier expert system. Differentiation Between Similar Species: In scenario A, a camera trap captures an image mistakenly identified by the global model as a hare (label 20) with 85% confidence. However, this image contains a rabbit. The global model’s initial output is then processed by an expert model specializing in smaller mammals, correctly identifying the rabbit (label 21) with a higher confidence of 96%. This demonstrates our specialized expert models’ effectiveness in refining the global model’s initial predictions, especially in distinguishing closely related species. Detection of Animals in Empty-Looking Images: In scenario B, the global model fails to detect a small, hidden animal (a mouse) and classifies the image as “background” (label 24). Then, the response is redirected to all expert models to re-evaluate the image. Each expert model provides a decision, and the final identification is determined through a voting system based on confidence levels. Ultimately, the expert model for micro-mammals correctly identifies the mouse with a confidence of 91%, showcasing our method’s robustness in detecting animals even in challenging, low-visibility conditions.

This work introduces a two-stage deep learning-based workflow approach (Fig. 2) aimed at automating mammal identification while addressing the primary challenges encountered in real-world environments. This novel strategy addresses the complexity of animal detection by employing a clustering approach based on groups of animals determined by species appearance similarity, generating expert models for each group and efficiently decomposing the problem into several simpler sub-problems. Rather than training a single model encompassing all animal species as different classes, independent models were trained for each animal group, enabling specialization in detecting animals with similar morphology. In this sense, the concluding phase integrates the global model, trained with all classes across four groups, which redirects its prediction to one of the four expert models to ascertain the outcome of animal detection. Expert models can achieve improved precision and generalization in detecting animals within their group, benefiting from fewer classes to learn from and enabling focused analysis of specific features and patterns associated with that particular group. This two-stage approach empowers us to capitalize on the unique strengths of individual expert models and enhance the final classification outcome based on their respective confidences.

This study analyzed a comprehensive five-year image dataset from Sierra de las Nieves and Doñana National Parks (Andalusia, Spain), comprising 24 mammal species. Our approach, distinct from the conventional deep learning classification methods, leverages an innovative animal clustering strategy combined with advanced computer vision techniques, specifically MegaDetectorV5^15^ based on YOLOv5^16^. This novel strategy outperforms traditional deep learning approaches in accuracy, particularly when locating the animal in the image is complex and distinguishing between species with subtle differences. Our results highlight the potential of combining clustering techniques with deep learning for enhanced object detection in complex natural environments.

## Background and related work

Artificial Intelligence (AI) tools have recently become increasingly important for detecting and identifying objects in images^17^. Object detection technologies rapidly expand within this approach to detect and localize animals within images or video frames^18^. Deep learning-based object detection algorithms are powerfully accurate in finding and locating items in photos, even with complicated backgrounds or when objects are partially obscured^19^ and allow automatic animal detection and identification, minimizing manual annotation requirements^20^. Moreover, a deep learning framework’s detection component operates more efficiently when anchor boxes are used to predict the location and size of objects in an image^21,22^. Object detection models focus on learning from the image section corresponding to the bounding box rather than the entire background. Furthermore, object detection models enhance the interpretability of the results, as they indicate the specific area of the image where the animal is detected. This information ensures that the model’s classification is based on the animal’s presence rather than the surrounding background. The current object detectors could be classified into one stage and two stages. One-stage detectors regard object detection as a regression or classification problem and use a unified framework to obtain the final categories and locations directly^23^, such as RetinaNet^24^, Single Shot Detector (SDD)^25^, AttentionNet4^26^ or You Only Look Once (YOLO). On the contrary, Two-stage detectors generate regions and classify each area to get different object categories, such as Regions with CNN features (R-CNN)^27^, Faster Region-based Convolutional Neural Network (Faster R-CNN)^28^ or Region-based Fully Convolutional Network (R-FCN)^29^. One-stage detectors are typically faster and are commonly used for real-time applications. Therefore, One-stage detectors may be more suitable for animal detection in camera trap photos because of the large number of images to process.

Automating animal identification in camera-trap images has been extensively explored e.g. EventFinder^30^ software for screening remotely captured images or ClassifyMe^31^ software for the identification of wildlife in camera trap images. Early works employed Traditional Machine Learning methods with hand-designed features for animal detection^32–34^. In^35^, sparse coding spatial pyramid matching (ScSPM) was used to extract local features from camera-trap images, and a linear Support vector machine (SVM) algorithm was employed for classification. This approach achieved 82% average classification accuracy on a dataset of over 7000 images encompassing 18 species. However, this approach is limited by its dependence on predefined characteristics, which may not adequately capture the extensive variability in wildlife images. Moreover, its labor-intensive nature impedes scalability and adaptability across diverse ecological settings. In contrast to previous approaches, our work aims to leverage object detection through deep learning to extract essential features for animal detection automatically.

Several recent studies have employed deep learning techniques for camera-trap image classification. For instance, in^36^, the authors introduced a two-step process based on Deep Convolutional Neural Networks (DCNN) to classify camera-trap images into three categories: human, animal, and background patches. Despite using a dataset of 30,000 images for validation, this approach was computationally slow and achieved a recall rate of 73.2%. A different method was proposed in^37^, utilizing a DCNN as a feature extractor to train traditional machine learning algorithms like K-nearest neighbors (KNN) and SVM for wildlife animal detection, achieving an accuracy of 91.4% on a standard camera-trap dataset. Although the dataset contained 20 animal species, each with around 100 image sequences, the classification was limited to animal or background classes. In^38^, the authors introduced an automatic computer vision-based species recognition method for camera-trap images. They compiled and annotated a standard camera-trap dataset comprising 20 common species found in North America, consisting of approximately 20,000 images. Despite their efforts, the achieved accuracy was only 38%, indicating considerable scope for improvement. Many studies have made use of the Snapshot Serengeti camera-trap dataset (SS hereinafter), which includes images of 48 animal species. In^14^, the authors assessed the capabilities of state-of-the-art deep neural networks (DNNs) on the SS dataset. They obtained an accuracy of approximately 57% (estimated from their plot, since the exact accuracy was not explicitly reported in the paper). Moreover, in^39^ authors tested DNNs to automatically extract information from images in the standard SS dataset. Remarkably, their method achieved an impressive overall accuracy of 93.8%. Despite these excellent general accuracy results, the study exhibits limitations in terms of performance when classifying rare classes. Similarly, in^40^ the smallest classes had the worst performance (0.18–0.32 F1-Score), while the classifier was robust (0.87–0.95) for highly represented classes. In this regard, one of the most significant issues is the data imbalance among animal species. For instance, in^39^, the class imbalance problem is approached by conducting data augmentation through oversampling and employing weighted loss techniques. The weighted loss method demonstrated the highest top-1 accuracy, with improved classification performance for some rare classes at the expense of reduced accuracy for more frequent classes. Moreover, the accuracy of the less represented classes increased significantly in some instances while showing no improvement for others. Another significant challenge encountered in animal image detection using camera traps lies in the discrimination between highly similar species (i.e., red deer and fallow deer) or among groups of species that appear in similar environmental settings and temporal characteristics. Distinguishing subtle differences between visually similar species is a challenge for both human annotators and machine learning algorithms, making accurate species classification difficult^41^. Even experienced biologists may struggle to differentiate between certain species, leading to ambiguous or subjective labeling^42^. In animal species classification, some previous work has focused on image classification tasks for which the animals’ species often differ only slightly in small details. For instance,^43,44^ work to distinguish different bird species, others such as^45^ focus on determining moth species with slight variance between classes. These works focus on techniques such as Fine-grained recognition^46^, which are part-based solutions that collect additional local information regarding attention or parts. However, these models are sensitive to variations in image quality, such as lighting conditions, angles, or occlusions, and do not generalize well to low-quality or complex images. In the same way, this challenge is underscored by recent findings^47^ that shed light on the intricacies associated with discerning among taxonomically related animals using image redundancy contained in sequences of images. This study highlights that the precision discrepancies between mouflon and goats are largely due to the inherent imbalance in the validation set, where the overrepresentation of chamois and sheep images significantly skews the classification results. Furthermore, challenges in identifying distinct features, such as ibex horns, contribute to the observed performance issues. Notably, the classification difficulties extend to nocturnal scenarios, where distinguishing between wolves, foxes, and dogs proves inherently challenging. Thus, this study elucidates the formidable obstacles associated with accurately classifying closely related species in the context of wildlife monitoring through camera traps.

## Materials and methods

Our work introduces a two-stage deep learning-based workflow to address the challenges encountered in the literature regarding animal detection from camera trap images. This approach involves an initial Grouping-based strategy for animal identification, which draws inspiration from field biology, where a generalist initially classifies most images and experts discern highly similar species, enhancing classification confidence^48^. The two-stage pipeline outperforms a single 24-class classifier by adopting a divide-and-conquer approach (Fig. 2). In the first stage, the global model classifies and redirects images to one of four expert models, each with a confidence score. The second stage involves the selected expert model performing its classification. This approach, focusing on fewer classes, improves precision and generalization by analyzing the distinct features of each group. By grouping similar species, we increased classification accuracy from 92 to 96.2% on a test dataset of over 120,000 images. This strategy enhances overall performance, addressing common issues such as misclassifying empty images and confusing similar species.

### Dataset description

The dataset used in this study was collected during fieldwork campaigns in Doñana National Park, Spain ($36^\circ 59'$ N $6^\circ 26'$W) where we deployed 58 camera traps along 2018–2022, and Sierra de las Nieves National Park ($36^\circ 44'$ N $4^\circ 59'$W), Spain where we deployed 33 cameras in 2022 (Fieldwork was conducted under permits: Doñana National Park 2018/18 and 2021/17; Sierra de las Nieves N. expt.:PNSN/AU/104-2021). Camera traps were attached to trees or wood sticks at a height of 0.5 m above ground in-game trails and passages where available or in open areas. Whenever a camera trap is triggered, typically in response to nearby animal movement, it captures a series of photographs. In Doñana, we used the camera model Browning Strike Force HD Pro, and in Sierra de las Nieves, we used a combination of Browning BTC-8E-HP5 Spec Ops Elite HP5 and Browning BTC-5HDPX Strike Force Pro (https://browningtrailcameras.com/). Additionally, we raised a call for images of species that were underrepresented in our data pull through the Spanish Society for the Conservation and Study of Mammals (SECEM (https://www.secem.es/)) and received complementary images from citizens and colleagues of several locations in Spain, those obtained with various camera models. The dataset employed in this study comprises 1,331,309 images encompassing 24 distinct species: wild boar *Sus scrofa*; feral cattle *Bos taurus*; feral horse *Equus caballus*; domestic sheep *Ovis orientalis aries*; domestic goat *Capra aegagrus hircus*; roe deer *Capreolus capreolus*; Iberian ibex *Capra pyrenaica*; European mouflon *Ovis aries musimon*; red deer *Cervus elaphus*; fallow deer *Dama dama*; red fox *Vulpes vulpes*, Egyptian mongoose *Herpestes ichneumon*, Eurasian badger *Meles meles*, feral dog *Canis lupus familiaris*; feral cat *Felis silvestris catus*; small-common genet *Genetta genetta*, Eurasian otter *Lutra lutra*; Iberian lynx *Lynx pardinus*; European wildcat *Felis silvestris*; stone marten *Martes foina*; rabbit *Oryctolagus cuniculus*; hare *Lepus granatensis*; mouse *Mus* or *Apodemus sp.*; rat *Rattus rattus*.

### Accelerating the annotation process of camera trap images

Deep learning algorithms based on object detection techniques have shown promising results in camera trap animal identification. However, the animal must be located and labeled inside the image to train these algorithms. Annotation includes tagging the image and generating bounding boxes, which involves enormous manual labor. In our research, we leveraged the capabilities of MegaDetectorV5^15^, based on the YOLOv5 architecture, as a semi-automatic labeling tool for detecting animals in camera trap images. Developed by Microsoft, this versatile object detection model is specifically designed to analyze extensive camera trap datasets. Trained on millions of images from diverse global environments, MegaDetector can identify three primary object classes: humans, animals, and vehicles. It also efficiently recognizes empty images that do not contain these specific classes. The use of MegaDetector substantially accelerates the annotation process, enabling biological experts to bypass the tedious manual task of locating animals within each image. Instead, experts can direct their efforts towards more nuanced aspects of data analysis. This semi-automatic approach to generate bounding boxes around detected animals streamlines the workflow, enhancing overall efficiency. In this sense, several research studies, such as^11,49,50^, have assessed the efficiency of MegaDetector in wildlife detection, e.g.,^11^ found that its use increased the processing speed by over 500%. The time required for the manual processing component was reduced by 8.4 times. In total, 596, 748 photos have been annotated with this semi-automatic labeling approach. The remaining images were labeled as *empty*, indicating the absence of animals. Moreover, the preparation of training data carefully accounted for the diverse range of environmental factors and capturing conditions of a real-world use case. Therefore, we aimed to make the data as diverse as possible, including photos from different times of day, seasons, weather conditions, lighting conditions, angles, and camera setups. This approach helps to enhance the model’s robustness and adaptability, ultimately leading to more accurate and reliable animal detection and classification in real-world camera trap settings.

### Grouping-based strategy for animal identification

We have developed an innovative clustering approach based on the animal’s appearance, which generates expert models for each group. Rather than training a single model encompassing all animal species as different classes, independent models were trained for each animal group, enabling specialization in detecting animals with similar morphology.

**Fig. 3:**
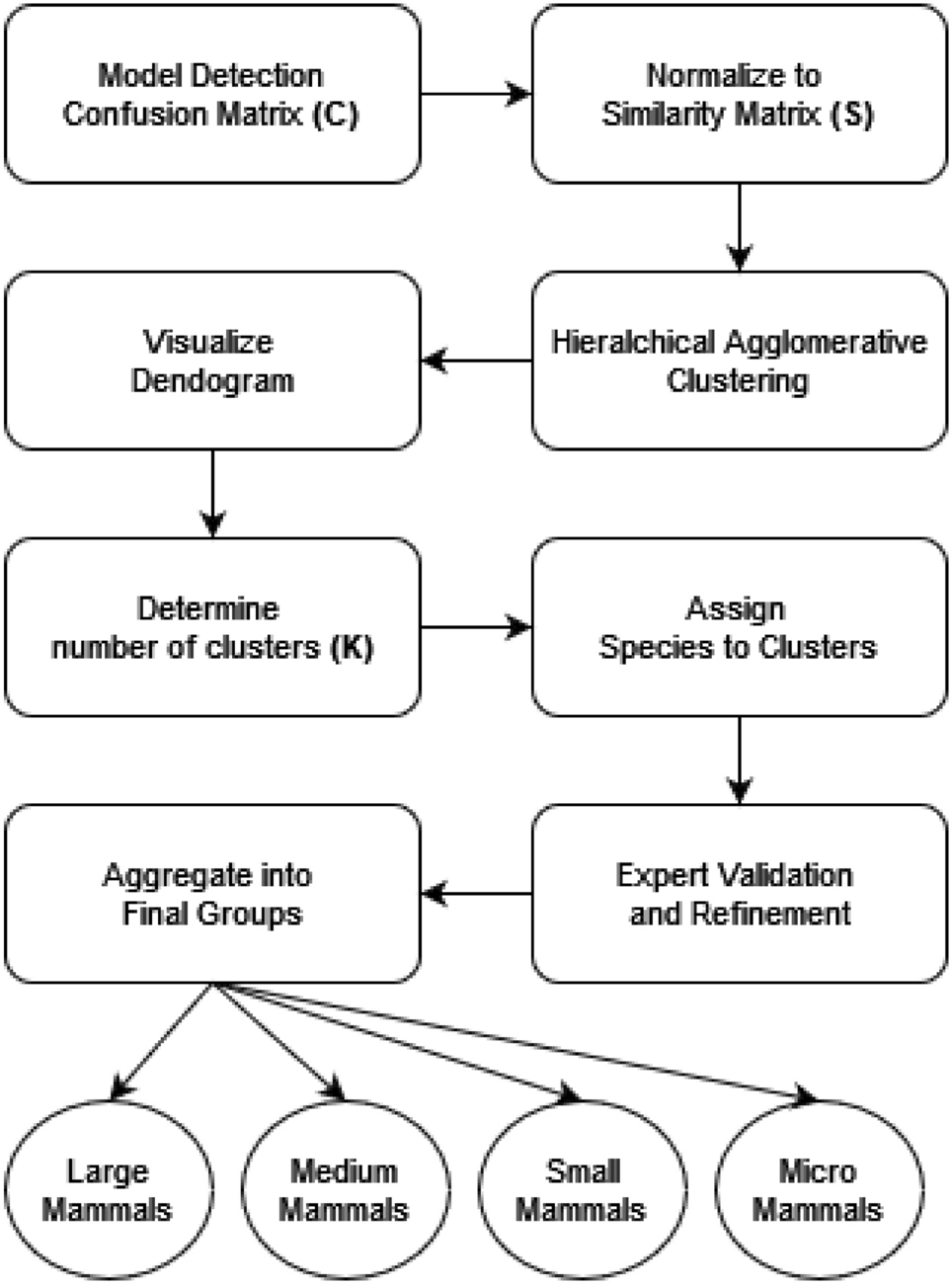
Schematic of the grouping-based strategy for animal identification.

We employed a systematic approach that integrates machine learning techniques to automate the grouping of animal species based on their detection similarities (Fig. 3).

The first step involves the construction of a confusion matrix, denoted as *C*, which captures the performance of our detection models by revealing the count of correct and misclassified predictions for each species.

Let *N* be the total number of species under consideration, and $C_{ij}$ represent the count of instances where species *i* was predicted as species *j*. To normalize this matrix and derive a similarity matrix, *S*, each element $S_{ij}$ is computed as:

1 $$\begin{aligned} S_{ij} = \frac{C_{ij}}{\sum _{k=1}^{N} C_{ik}}. \end{aligned}$$

The resulting *S* matrix represents the normalized similarity between species, where values close to 1 indicate high similarity and values close to 0 indicate dissimilarity.

With the similarity matrix in hand, we applied hierarchical agglomerative clustering to group species accordingly. The linkage matrix resulting from this process provides insights into the hierarchical structure of species relationships. By visualizing the dendrogram derived from the linkage matrix, we obtain a tree-like representation, where closely related species happened to appear in proximity.

To facilitate interpretability and application, we determined the number of clusters (*K*) based on the dendrogram structure four in our case. Subsequently, the agglomerative clustering algorithm was employed to assign each species to one of the *K* clusters. This process results in an assignment vector $\textbf{A}$, where $A_i$ represents the cluster to which species *i* is assigned.

It is remarkable that this automated process produced clusters that perfectly aligned with the species’ biological characteristics, such as taxonomy, size, behavior, and ecological roles and where therefore validated by the biologists:

1. Large mammals: Order Perissodactyla (Fam. Equidae) and Order Artiodactila (Fam. Bovidae and Suidae)
2. Medium mammals: Order Carnivora (Fam. Canidae and Felidae)
3. Small mammals: Order Carnivora (Fam. Mustelidae, Viverridae, and Herpestidae)
4. Micromammals: Order Lagomorpha (Fam. Leporidae) and Order Rodentia (Fam. Muridae)

### Deep learning architectures

In this work, various state-of-the-art Deep Neural Networks were tested and compared to identify the highest-performing networks. Specifically, deep learning models focused on image classification, such as GoogLeNet^51^, AlexNet^52^, ResNet50^53^, ResNet101^53^, ResNet152^53^, VGG16^54^, VGG19^54^, and Xception^55^, were evaluated. Additionally, deep learning-based object detection models, including YOLOv5, YOLOv5 (MegaDetector), YOLOv8, and Faster R-CNN, were also tested. Each deep learning model was trained with all animal classes in the training set and evaluated using the F-score metric (eq 3), which balances precision and recall (Eq. 2).

2 $$\begin{aligned} precision = \frac{TP}{TP + FP} \nonumber \\ \quad recall = \frac{TP}{TP + FN} \end{aligned}$$

3 $$\begin{aligned} F1-score = \frac{2 * precision * recall}{precision + recall} \end{aligned}$$

Precision measures the accuracy of positive predictions, while recall measures the ability to identify all relevant instances. The F-score, as the harmonic mean of precision and recall, comprehensively evaluates the model’s performance. Relying solely on accuracy can be misleading, particularly with imbalanced datasets, as it may overemphasize dominant classes and neglect rare or endangered species. The F-score offers a more nuanced assessment, ensuring that performance across all classes is fairly evaluated and that more frequent ones do not overshadow the detection of less common species. This ensures a robust evaluation of the model’s effectiveness in real-world applications.

As shown in Table 1, the best results were obtained using YOLOv5 and MegaDetector as pre-trained models, achieving an F-score of 92%. Transfer Learning^56^, a widely adopted technique, enables the reuse of knowledge gained from one task to serve as a starting point for training models on related tasks. Accordingly, we used MegaDetector as a pre-trained model in our two-stage pipeline, leveraging the extensive knowledge it acquired from diverse training on a wide range of animal species and ecosystems. More details about model architecture and hyperparameters can be found in Appendix A: Experimental settings.

**Table 1:** Accuracy of different deep learning models for animal species classification. YoloV5 (MegaDetector) obtained the best metrics (highlighted in bold).

Deep learning model	F-score
GoogLeNet	85.5%
AlexNet	87.3%
ResNet50	88.3%
ResNet101	85.7%
ResNet152	85.2%
VGG16	80.7%
VGG19	81.7%
Xception	91.0%
Yolov5	89%
Yolov5 (MegaDetector)	92%
Yolov8	91%
Faster RCNN	90%

### Enhancing animal detection with a two-stage pipeline using expert models

Finally, a global model was trained with all animal classes and four expert models for each group of animals. Table 2 shows how each expert model achieves improved metrics on the validation sets by reducing the number of species per model and considering similar species within each model. Overall, the expert models exhibit superior animal detection and classification performance in the images. The global model tends to fail in classifying large mammals. This is often because these animals appear close to the camera trap, resulting resulting in only a small part of the body being visible. Additionally, the global model struggles with detecting micromammals, as these animals are typically difficult to spotin the images due to their small size.

**Table 2:** Metrics results during the training phase.

Model	mAP@0.5	Precision	Recall
Large mammals	0.91	0.93	0.91
Medium mammals	0.97	0.95	0.94
Small mammals	0.97	0.95	0.95
Micromammals	0.98	0.96	0.96
Global model	0.91	0.92	0.92

## Results

**Fig. 4:**
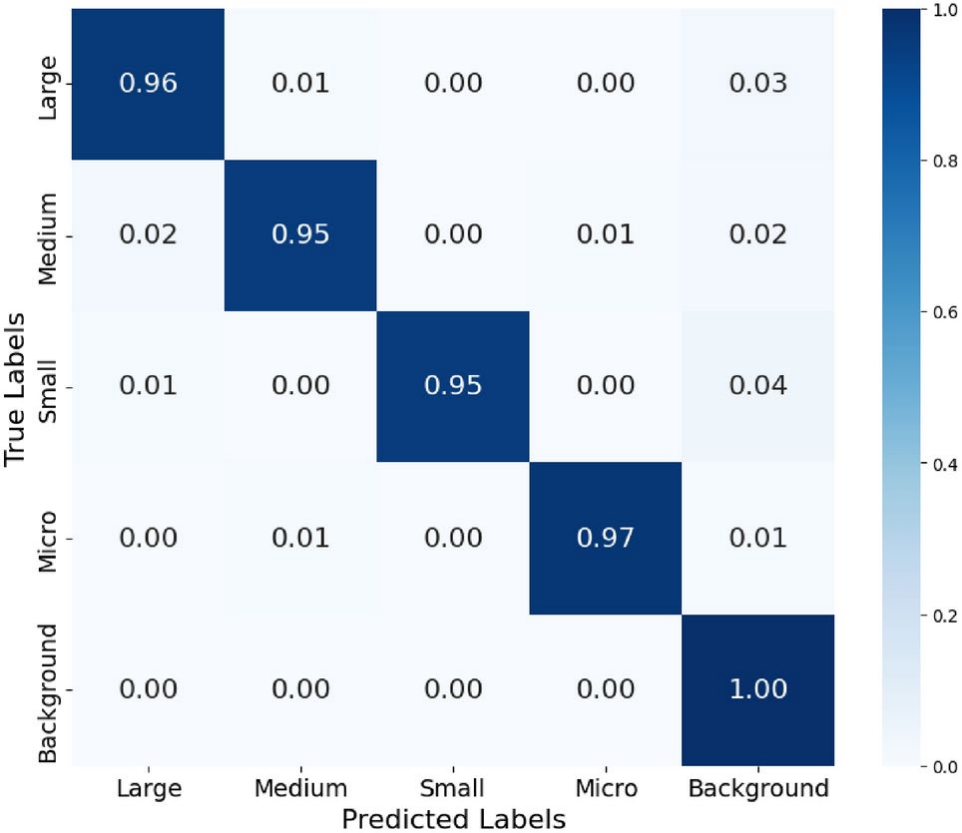
The confusion matrix illustrates the performance of the global model in classifying animal species, which subsequently redirects the response to one of the expert models for final classification.

A series of experiments were conducted to demonstrate the feasibility of our proposal on an out-of-sample test dataset containing over 120,000 images. These images were taken in contexts that were never encountered during the training stage. In the first stage of the methodology, the global model classifies images into four groups, obtaining an impressive F-score of 97% (Fig. 4). This high performance is due to the ease with which the global model can distinguish between these four major groups of animals, whose appearance characteristics are significantly different. In the second stage, the decision from the global model is redirected to the expert models, achieving a final F-score of 96.2%.

Next, we will showcase how our approach achieves enhanced accuracy in addressing the challenges of animal detection in real-world scenarios. We will compare the metrics obtained from a single-stage pipeline, which uses a global model trained on all classes, with the metrics from our two-stage pipeline (Table 3). Our two-stage approach demonstrates significant improvements in accuracy due to the use of models customized for groups of animals with similar morphological characteristics. For a more detailed view of the results, see Appendix B: Two-stage pipeline evaluation.

**Table 3:** Comparison of evaluation results between the global YOLOv5 model using MegaDetector as a pre-trained model and our two-stage pipeline on an out-of-sample test dataset containing over 120,000 images. These images were taken in contexts that were never encountered during the training stage. The final F1-Score of the global model is 0.92, while our two-stage pipeline achieved an F1-Score of 0.962.

Class	Single-stage metrics			Two-stage metrics			Support
	Precision	Recall	F1-score	Precision	Recall	F1-score	
Background	0.90	1.00	0.95	0.96	1.00	0.98	23417
Feral cattle (bos)	0.92	0.85	0.88	0.95	0.92	0.94	6613
Domestic goat (caae)	0.94	0.88	0.91	0.97	0.95	0.96	7535
Roe deer (caca)	0.41	0.95	0.57	0.93	0.97	0.95	97
Feral dog (can)	0.96	0.94	0.95	0.95	0.99	0.97	4558
Capra pyrenaica (capi)	0.95	0.93	0.94	0.98	0.98	0.98	7736
Red deer (cer)	0.91	0.81	0.86	0.95	0.90	0.92	7197
Fallow deer (dam)	0.24	0.92	0.38	0.73	0.94	0.82	232
Feral horse (equ)	0.90	0.87	0.88	0.94	0.94	0.94	6915
European wildcat (fel)	0.54	0.97	0.70	0.89	0.97	0.93	128
Feral cat (fsi)	0.99	0.97	0.98	0.99	0.98	0.99	6068
Small-common genet (gen)	0.99	0.96	0.98	0.99	0.98	0.99	5240
Egyptian mongoose (her)	0.95	0.97	0.96	0.98	0.98	0.98	3967
Hare (lep)	0.96	0.93	0.95	0.98	0.95	0.97	4158
Eurasian otter (lut)	0.83	0.95	0.88	0.96	0.98	0.97	389
Iberian lynx (lyn)	0.67	0.87	0.76	0.88	0.97	0.93	61
Stone marten (mafo)	0.99	0.97	0.98	0.99	0.99	0.99	7689
Eurasian badger (mel)	0.82	0.96	0.88	0.94	0.99	0.96	1300
Mouse (mus)	0.99	0.88	0.93	0.99	0.97	0.98	3283
Rabbit (ory)	0.67	0.91	0.77	0.92	0.96	0.94	674
Domestic sheep (ovar)	0.95	0.91	0.93	0.99	0.95	0.97	7468
European mouflon (ovor)	0.58	0.91	0.71	0.91	0.99	0.95	572
Rat (rara)	0.80	0.96	0.88	0.98	0.98	0.98	342
Wild boar (sus)	0.94	0.90	0.92	0.96	0.95	0.95	7421
Red fox (vul)	0.94	0.91	0.93	0.93	0.96	0.94	7502

### Challenge 1: Dealing with background

Most models implemented using a one-stage pipeline tend to misclassify many images as empty, particularly in challenging conditions such as low light, nighttime, or when the animal is partially concealed.

In this scenario (Fig. 2 scenario B), when the global model classifies the image as “background”, the response is redirected to all expert models, which then make the final decision based on their confidence rates. This scenario is the most complex in the decision-making process, as it is where the global model tends to have a higher error rate (see global confusion matrix). There are two crucial situations in these cases:

- True Positive (Background): The global model predicts “background,” and the image is indeed empty. In this case, the global model redirects the response to all four expert models, which also predict “background” 100% of the time.
- False Negative (Animal): The global model predicts“background,” but the image actually contains an animal. Here, the response is redirected to the four expert models. Typically, all expert models detect the presence of an animal, even if it does not belong to their respective groups. The final decision is made by the expert model with the highest confidence rate. The model containing the animal usually has a very high confidence rate, while the other three models, which do not contain the animal in their groups, typically have confidence rates below 60%.

This approach has demonstrated the capability to identify the animal in the image in over 99% of cases, including those where the animal is hidden far in the background, such as large mammals like *feral cattle (bos)*, *red deer (cer)*, and *feral horse (equ)*, or micromammals like *mouse (mus)* that are very difficult to identify due to their small size. See Appendix B: Two-stage pipeline evaluation for more details.

### Challenge 2: Dealing with unbalanced datasets

The dataset is heavily unbalanced, with some species being much more frequent than others. This imbalance poses challenges for machine learning techniques, which tend to be biased towards classes with more examples. Consequently, the model may primarily predict the more frequent types, such as *wild boar* or *red deer*, achieving high accuracy without effectively learning the less common classes. This is particularly concerning when less frequent classes like the *Iberian lynx* hold greater scientific interest and conservation importance. Addressing class imbalance is, therefore, crucial to ensure comprehensive and accurate ecological studies.

The Grouping-Based Strategy mitigates class imbalance by reducing the number of animal classes each expert model needs to handle. Notably, animals with similar characteristics often exhibit analogous patterns, resulting in comparable appearances in camera trap images. For instance, larger mammals tend to have a higher frequency of occurrences in the dataset than smaller mammals. Thus, our strategy, grounded in animal grouping, presents a promising solution to the inherent challenge of imbalanced data. As an example, our approach has improved the F-score of minority classes (see Table 3), such as the *Iberian lynx (lyn)* from 76% to 93%, *roe deer (caca)* from 57 to 95% , *fallow deer (dam)* from 38 to 82% or *European wildcat (fel)* from 70 to 93%.

It is important to note that class balancing strategies were implemented during training for each animal group model since there were some less-represented species within each group. However, these data-balancing strategies become more straightforward and effective as the number of classes decreases. To address this imbalance, we modified the YOLO configuration file (typically in YAML format) to assign higher weights to underrepresented species in the dataset. Additionally, we applied data augmentation techniques targeted at the most imbalanced species. This approach helped improve overall performance and mitigated the risk of the model becoming biased towards species with larger sample sizes.

### Challenge 3: Dealing with similar animal species

Most misclassifications in one-stage models trained with all classes occur among very similar species, such as *roe deer* and *fallow deer*, *rabbit* and *hare*, or *Iberian lynx* and other felines. Additionally, misclassifications occur with some larger mammals (e.g., *feral cattle*, *feral horse*), particularly in night images where species identification is inherently challenging or when only a small portion of the animal, such as a leg or hoof, is visible.

Thanks to the grouping based on appearance and size, we can develop more specialized models that mitigate the bias from highly similar species. By clustering animals with similar physical characteristics, our approach allows each expert model to focus on a narrower set of species, enhancing its ability to differentiate between them accurately. This specialization reduces the confusion often observed in models trained on a wide variety of species.

For instance, when species such as *red deer and* *fallow deer* are grouped based on their size and appearance, the expert model can fine-tune its parameters to recognize subtle differences that a general model might overlook, increasing the F-score from 86 to 92% for *red deer* and from 38 to 82% for *fallow deer*, as shown in Table 3. Similarly, by clustering smaller animals like rabbits and hares, the F-score improved from 77 to 94% and 95 to 97%, respectively. For larger mammals such as *feral cattle* and *feral horse*, the F-score increased from 88 to 94% for both species. Each model becomes adept at identifying species’ unique features within its group.

This targeted approach improves the overall accuracy of species identification and addresses the common issue of misclassification in one-stage models. It ensures that the specialized models maintain high performance even in challenging conditions, such as night images or when only a part of the animal is visible. By reducing the workload on a single, all-encompassing model, our strategy promotes more reliable and precise animal detection across diverse ecological studies. For a more detailed view of the results, see Appendix B: Two-stage pipeline evaluation.

## Discussion

Our study introduces the first AI workflow for object detection in camera trap images that employs a serial approach, combining a general model followed by expert models tailored for specific animal groups. While current deep learning models have significantly improved detection capabilities, developing algorithms that perform well across various classes remains challenging. Most works in this area extend multi-class models to handle multiple types, but these models often need more flexibility when accommodating new object descriptors. Furthermore, they typically do not address multiple criteria simultaneously. Some approaches rely on iteratively executing single-class models for each target class, which results in linear scaling of training and run times. Our serial approach has immediate applicability to solve real-world challenges in camera-trap scenarios, and its main advantages for producing higher-quality results are:

- Reducing the data unbalance problem. Clustering animals in groups reduces the significance of sample differences between classes.
- Reduce misclassification among similar species. By training expert models for specific animal groups, the models can focus on more subtle details that allow for distinguishing similar species. This is a highly relevant result because classifying animal species correctly is crucial. Misclassification can result in erroneous fauna inventories and biases in estimating animal species’ geographic range or habitat use, negatively affecting scientific studies’ reliability and hindering conservation and management efforts^48^. Misidentification is common among phenotypically similar species, even for trained human observers^42,48^. For example,^42^ found that none out of ten human observers assessed for identifying animals in camera trap images identified all mammalian wildlife across the sub-datasets and that the majority of misidentification happened between similar species (two African rhinoceros species, two zebra species as well and two similar species of dwarf antelopes (n = 12, 52.2%). Similarly,^48^ found that experts were sometimes even inconsistent with themselves, providing different classifications for similar species (bobcat or lynx) over the same images shown in two trials separated by ten weeks.
- Reduce the number of false negatives. Expert models can learn subtle characteristics of the animals belonging to specific groups.^42^ found that the species missed mainly by human observers were small mammals (56.5%) with body masses < 5 kg. For example, it is common that the model fails to detect mouse in the image, as these animals are often well-hidden and typically appear in photos taken at night. However, independent small mammal models can detect mouse even in complicated situations.

Although there are many studies on multi-class animal species detection, creating algorithms that work well with multiple species remains challenging. Designing efficient multi-class species detection systems is a complex and active research area, as the scale of species classes varies depending on the tasks. Our two-stage pipeline can be a solution for creating a scalable multi-class model with many classes.

Creating a global model to distinguish between groups of animals is both straightforward and effective. The model’s performance and taxonomic coverage suggest its potential for automatically sorting vast quantities of images across various taxonomic groups. This capability is particularly valuable in studies examining the impact of anthropization on large mammal communities^47^. Furthermore, our semi-automatic clustering approach for developing expert models for each animal group reduces the workload on a single model with a high number of classes, resulting in promising improvements in overall performance.

### Limitations and future work

The methodology presented here is based on algorithms trained using manually labeled images. Therefore, any human error in the classification used as“truth” would be introduced to the AI workflow. To avoid or minimize it, we emphasize the importance of using expert and multiple observers, as recommended by^42,48^ and that these observers discard the images in the training dataset where species cannot be confidently classified. Our approach has room for improvement in future developments. In the current case, the four groups into which all species were divided were created using our grouping-based strategy, with the number of groups decided by expert biologists. This could be accomplished in the future using other clustering algorithms based on Artificial Intelligence, allowing for a fully automatic methodology that can be extrapolated to other scenarios with different species compositions.

## Figures and Tables

**Fig. 5:**
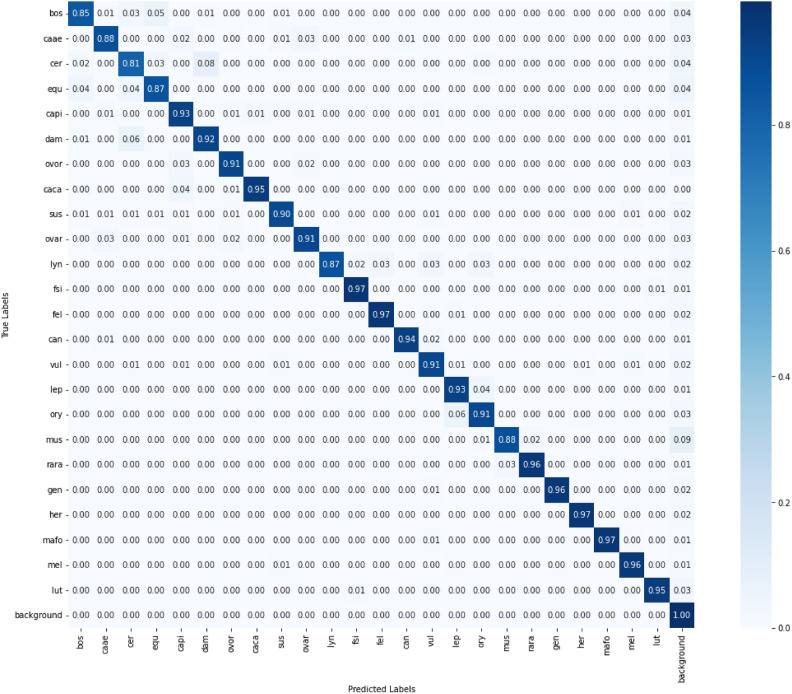
Confusion matrix of the single-stage pipeline, where the global model, YOLOv5 (MegaDetector), is trained with all animal classes

**Fig. 6:**
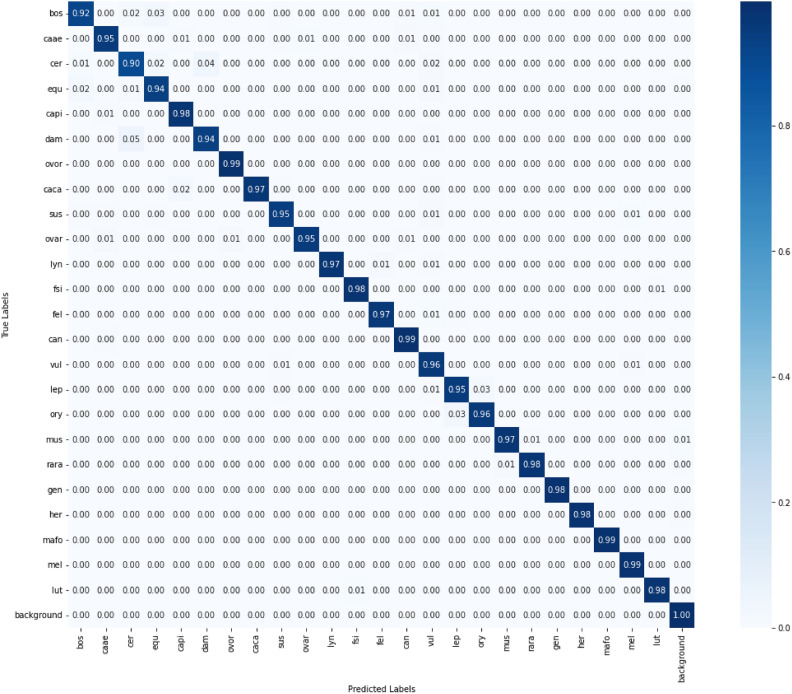
Confusion matrix of our two-stage pipeline based on expert models, which leverages specialized expert models for animal species detection. The global model, YOLOv5 (MegaDetector), is trained with all animal classes, while the expert models are trained on specific groups identified as large, medium, small, and micro mammals. The confusion matrix demonstrates the enhanced performance and accuracy of our two-stage pipeline in detecting various animal species

**Fig. 7:**
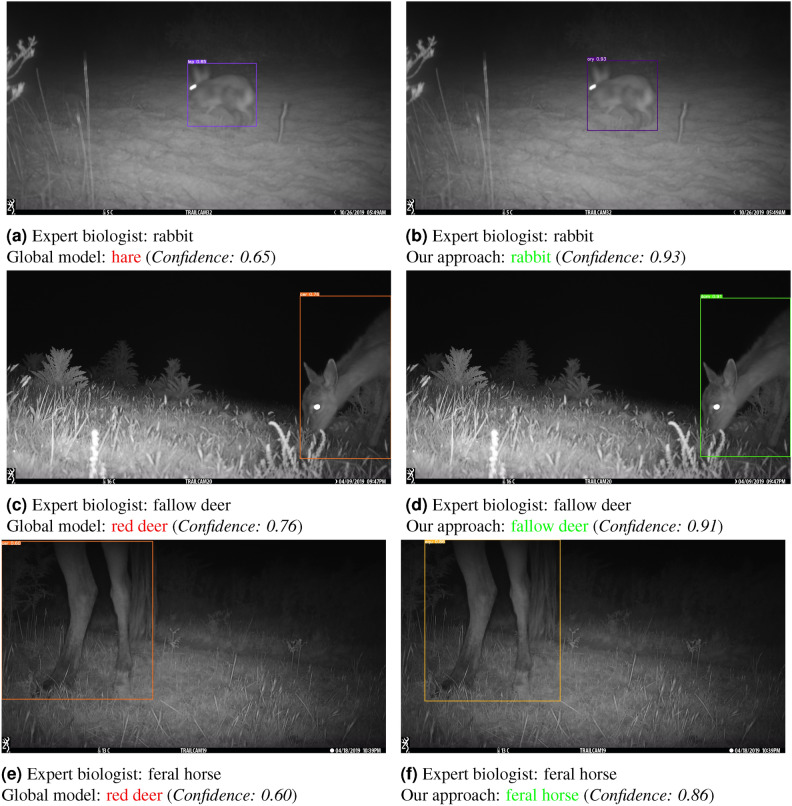
Examples of challenging animal detection in camera trap images where the global model misclassifies animal species. The model sometimes confuses the animal due to species similarity combined with low-light conditions. At the same time, in other situations, it misclassifies the animal because only a part of it, such as a leg, is visible. However, our two-stage approach based on expert models has successfully corrected these predictions by leveraging specialized knowledge for each group of animals

**Fig. 8:**
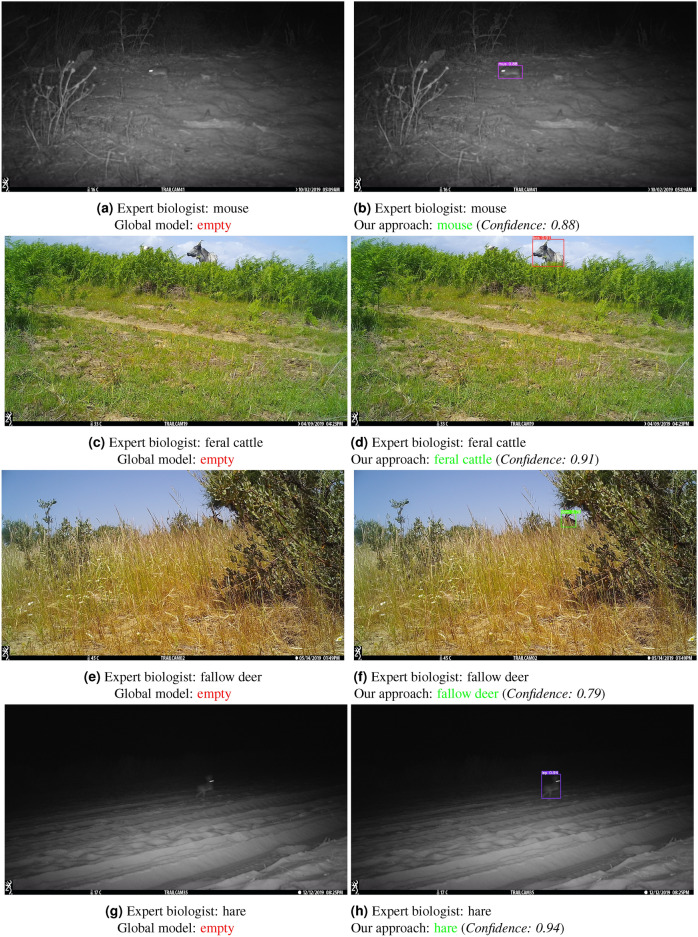
Left: situations where the global model fails to detect the animal in the images due to challenging conditions such as darkness, proximity of the animal to the camera, or the animal being camouflaged against the background. Right: same situations where the expert models of our two-stage pipeline have successfully corrected these issues, accurately detecting the animal

## Data Availability

The datasets generated and/or analyzed during the current study are not publicly available due to its large volume, but are available from the corresponding author on reasonable request.

## Appendix

### Appendix A: Experimental settings

Transfer learning is a powerful technique that leverages knowledge gained from one task and applies it to a different, related study. In particular, we used GoogLeNet, AlexNet, ResNet50, ResNet101, ResNet152, VGG16, VGG19, and Xception as pre-trained models on the ImageNet dataset, which contains 1.3 million images from 1,000 classes of man-made and natural images^57^. These models were employed to extract high-level features from the images. Subsequently, we trained a classifier on top of these features using our dataset to classify all animal species in Doñana and Sierra de la Nieves study areas.

We trained the networks via backpropagation using Stochastic Gradient Descent (SGD) optimization with momentum and weight decay. Each model underwent training for 100 epochs, starting with an initial learning rate of 0.01. The model was checkpointed after each epoch, and the results of the most accurate model on the expert-labeled test set were reported. The settings for these experiments are detailed in *Deep Learning Classification* Table 4. Similarly, we configured the training parameters for the deep learning object detection models, including YOLOv5, YOLOv5 (MegaDetector), YOLOv8, and Faster R-CNN, as shown in *Deep Learning Detection* Table 4.

Finally, we used YOLOv5 with the MegaDetector model as a pre-trained model. The MegaDetector model has been trained on a large dataset comprising millions of images captured across diverse locations worldwide. For our specific task, we used the MegaDetector model as a starting point, initializing the weights of our models for further training on our dataset. By freezing the model’s first ten layers (layers 0-9), representing the backbone architecture, we can extract high-level features from the millions of camera trap images. These features are then used to classify our animal species, effectively taking advantage of the rich knowledge learned by the MegaDetector model during its extensive training on a diverse range of ecosystems. Deep Learning detection models were trained using backpropagation, implementing Stochastic Gradient Descent (SGD) optimization with momentum and weight decay. Each model underwent training for 300 epochs, starting with an initial learning rate and a weight decay policy, as shown in Table 4. To ensure robustness, we used a batch size of 128 with an image size of 1280, facilitating effective batch normalization of the input images. Throughout the training process, we took periodic snapshots of the model after each epoch, enabling us to track its progress. Additionally, we implemented a model checkpoint with patience of 50 epochs, meaning that if the model’s accuracy did not improve within 50 epochs, we reverted to the best-performing model. Finally, we evaluated and reported the results of the most accurate model based on its performance on the validation set. This approach allowed us to identify and select the best model for further analysis and evaluation.

**Table 4:** Training configuration parameters for deep learning classification and detection models.

Settings	Classification	Detection
Optimization	SGD	SGD
Momentum	0.9	0.9
Weight decay	0.0005	0.0005
Number of epochs	100	300
Learning rate	0.01	0.01
Batch size	64	128
Image size	256	1280
Batch normalization	Applied	Applied
Model checkpoint	Each epoch	Each epoch
Patience	20 epochs	50 epochs
Animals species	24	24

### Appendix B: Two-stage pipeline evaluation

Measured on the labeled test set, the global model achieved an F1-score of 0.92%. Despite achieving good results, we have identified several challenges that must be addressed, as shown in Fig. 5. The model accurately classifies images without animals as background images, which makes sense given the pre-trained MegaDetector model. However, the model struggles to detect certain animals in specific situations, such as smaller animals like mouse. Furthermore, the model tends to confuse similar animal species, such as deer and roe deer or hare and rabbit.

Our two-stage pipeline leverages the strengths of both a global model and specialized expert models. The confusion matrix of our two-stage pipeline, shown in Figure 6, demonstrates significant improvements in accuracy and classification performance.

- The specialized expert models are trained to focus on specific groups of animals, allowing for better detection of smaller species such as mouse. The system enhances the identification accuracy of these species by redirecting the classification task to an expert model that is fine-tuned for small mammals.
- Our approach reduces the confusion between similar species by employing expert models specializing in specific morphological characteristics. For example, red deer and fallow deer, and hare and rabbit are more accurately classified by models trained on features specific to each group. This specialization minimizes misclassification errors and improves overall precision.
- The two-stage pipeline mitigates the confusion caused by shared characteristics among different species. The global model’s initial classification step ensures images are directed to the appropriate expert model. It can then use its specialized knowledge to distinguish between species that may appear similar when only partial features are visible, such as the legs of cows and horses.
- While the global model performs well in identifying background images, the two-stage approach further refines this by using the confidence scores from expert models to confirm the absence of animals. This ensures that images are accurately classified as background, reducing false positives.

Moreover, Figs. 7 and 8 illustrate examples where our two-stage pipeline based on expert models significantly improves animal species classification. In these challenging scenarios, the global model struggles due to low-light conditions, partial animal visibility (e.g., only a leg is visible), or high similarity between species. However, our two-stage approach successfully addresses these issues. By leveraging specialized expert models, it accurately identifies the species even under these challenging conditions, demonstrating its robustness and effectiveness.
